# Acute Co-Ingestion of Caffeine and Sodium Bicarbonate on Muscular Endurance Performance

**DOI:** 10.3390/nu16244382

**Published:** 2024-12-19

**Authors:** Juan Jesús Montalvo-Alonso, César Munilla, Laura Garriga-Alonso, Carmen Ferragut, David Valadés, Paola Gonzalo-Encabo, Alberto Pérez-López

**Affiliations:** Departamento de Ciencias Biomédicas, Área de Educación Física y Deportiva, Facultad de Medicina y Ciencias de la Salud, Universidad de Alcalá, 28801 Madrid, Spain; jesus.montalvo@uah.es (J.J.M.-A.); carmen.ferragut@uah.es (C.F.); david.valades@uah.es (D.V.)

**Keywords:** sport nutrition, ergogenic aids, caffeine, sodium bicarbonate, resistance exercise, load-power relationship

## Abstract

**Background:** Caffeine and sodium bicarbonate individually enhance muscular endurance by delaying fatigue, but their combined effects have scarcely been studied. **Objectives**: This study aimed to evaluate the acute effects of co-ingesting caffeine and sodium bicarbonate on muscular endurance at different loads in bench press and back squat exercises. **Methods**: Twenty-seven recreationally trained participants (female/male: 14/14; age: 23 ± 3.6 years) were randomized to four conditions in a double-blind, crossover design: (a) sodium bicarbonate and caffeine (NaHCO_3_ + CAF); (b) sodium bicarbonate (NaHCO_3_); (c) caffeine (CAF); (d) placebo (PLA); ingesting 0.3 g/kg NaHCO_3_, 3 mg/kg caffeine or placebo (maltodextrin). Participants performed two muscle endurance tests on bench press and back squat exercises at 65% and 85% 1RM, performing as many repetitions as possible in one set until task failure. **Results**: CAF increased the number of repetitions (*p* < 0.001; η_p_^2^ = 0.111), mean velocity (V_mean_, *p* = 0.043, η_p_^2^ = 0.16), and mean power output (W_mean_, *p* = 0.034, η_p_^2^ = 0.15) compared to placebo. These effects were observed in back squat exercise at 65%1RM in V_mean_ (3.7%, *p* = 0.050, g = 1.144) and W_mean_ (5.2%, *p* = 0.047, g = 0.986) and at 85%1RM in V_mean_ (5.4%, *p* = 0.043, g = 0.22) and W_mean_ (5.5%, *p* = 0.050, g = 0.25). No ergogenic effects were found in NaHCO_3_ + CAF) or NaHCO_3_ conditions. **Conclusions**: CAF increased muscular endurance performance in male and female participants by increasing the number of repetitions, mean velocity, and power output; however, when NaHCO_3_ was ingested, these effects were not detected.

## 1. Introduction

Caffeine and sodium bicarbonate (NaHCO_3_) are widely used supplements by athletes in various sports, such as swimming or cycling, due to their ability to enhance endurance capacity [1,2]. Since caffeine and NaHCO_3_ act through different physiological mechanisms [2,3,4], it is plausible that co-ingesting them could result in an additive ergogenic effect. Despite this potential, research examining the combination of these supplements in the context of muscular endurance remains limited.

Caffeine and NaHCO_3_ have improved performance in exercises lasting 30 s to 8 min [1,2]. Caffeine works by antagonizing adenosine receptors (A_1_ and A_2A_) in the central nervous system (CNS), which leads to reduced fatigue and perceived exertion, increased alertness and vigor, and enhanced recruitment of muscle fibers during contractions [2]. Additionally, caffeine may facilitate calcium (Ca^2+^) mobilization, increasing muscle ability to produce force and delaying fatigue by preventing a decline in Ca^2+^ availability [5]. On the other hand, NaHCO_3_ ergogenic effects occur through enhanced extracellular buffering, leading to increased extracellular pH and base excess, which assists in removing H^+^ and lactate from working muscles [6]. This mechanism improves intramuscular pH, promoting higher glycolytic rates [6], faster ATP resynthesis [7,8], and better utilization of Ca^2+^ to sustain performance and delay fatigue [3,4].

Although both caffeine and sodium bicarbonate (NaHCO_3_) have been shown to individually enhance exercise performance [1,2], their combined effects remain underexplored. Muscular endurance, defined as the ability of a muscle or muscle group to maintain force output or perform repeated contractions against resistance over time, has been investigated following isolated caffeine and NaHCO3 supplementation. Research on caffeine has examined a range of loads from 35% to 85% of one-repetition maximum (1RM) and various protocols, including single sets, multiple sets, and drop-sets, often reporting performance improvements [9,10]. However, the magnitude of these improvements appears to depend on the type of exercise, with lower-body movements, such as squats, showing greater benefits compared to upper-body exercises like the bench press [11]. Physiologically, caffeine’s ability to mitigate fatigue-related reductions in force production further highlights its potential to enhance muscular endurance [12].

Similarly, NaHCO_3_ has been shown to enhance muscular endurance by buffering the accumulation of H^+^ during exercise [13]. Studies using a wide range of loads (from 20% to 80%) and various protocols (e.g., single and multiple sets) have yielded conflicting results regarding NaHCO_3_’s efficacy in improving muscular endurance [14]. Some studies have found no significant performance enhancement following the intake of 0.3–0.4 g/kg of NaHCO_3_ 60 to 90 min before exercise [15], while others have reported notable improvements [16]. A meta-analysis by Grgic et al. [14] concluded that a dosage of 0.3 g/kg NaHCO_3_, taken 60 to 180 min prior to exercise, could enhance muscular endurance, though not muscular strength.

Despite the available evidence on the individual effects of these supplements, no research has investigated their combined impact on muscular endurance. It is plausible thought that the co-ingestion of caffeine and NaHCO_3_ may promote additive ergogenic effects delaying muscular fatigue. The intramuscular accumulation of H^+^ caused by extenuating exercise would increase the competition between H^+^ and Ca^2+^ at the troponin-biding [17], inhibiting PCr resynthesis [18] and phosphofructokinase activity [6]. Thus, we hypothesized that the buffering capacity of NaHCO_3_ would facilitate the ability of caffeine to stimulate intramuscular Ca^2+^ availability and utilization, together with an improved ATP resynthesis, allowing the synergic ergogenic effect of the co-ingestion of NaHCO_3_ and caffeine. Consequently, the aim of this study was to evaluate the acute effects of co-ingesting caffeine and NaHCO_3_ on muscular endurance performance in resistance-trained males and females, at different loads in the bench press and back squat exercises.

## 2. Materials and Methods

### 2.1. Participants

A sample size of 24 participants was deemed adequate to compare the effects of the four supplement conditions and two exercise types across the two measurement points (65% and 85% 1RM), with an effect size of 0.3 (α = 0.05; 1 − β = 0.80) (v3.1, G*Power, Dusseldorf University, Düsseldorf, Germany) [19]. Finally, twenty-eight participants (age: 23 ± 4 years; sex ratio: 14 females and 14 males) completed the study. All participants were resistance-trained, with an average of 2.6 ± 1.2 years of structured resistance training experience. They trained their upper body 3.0 ± 1.4 days/week and their lower body 2.5 ± 1.6 days/week. The average 1-repetition maximum (1RM) relative per body mass was 0.99 ± 0.24 for the bench press and 1.40 ± 0.29 for the back squat. Participants’ dietary caffeine intake was 8.48 ± 8.0 mg/kg/day, as determined by a 24 h dietary recall. Eight out of the fourteen female participants began the trial during the follicular phase of their menstrual cycle.

To participate in the study, individuals had to meet the following criteria: (a) age between 18 and 35 years of age; (b) have no history of neuromuscular, musculoskeletal, neurological, immunological, or cardiometabolic disorders; (c) demonstrate the ability to perform back squats and bench press exercises with loads equivalent to at least 100% and 80% of their body weight, respectively; (d) possess a minimum of six months of prior resistance training experience and have trained at least three times per week over the past three months, as verified via questionnaire; and (e) refrain from using any medications, drugs, stimulants, or sports supplements throughout the duration of the study.

Before participation, all individuals were fully informed of the study’s procedures, potential risks, and any associated discomforts, after which they provided written consent. The study protocol adhered to the ethical principles outlined in the Declaration of Helsinki and received approval from the University Ethics Committee.

### 2.2. Experimental Design

The study employed a double-blind, placebo-controlled, randomized crossover design. Participants attended the laboratory on five different occasions. During the first visit, their body composition, dietary habits, and physical activity levels were evaluated. Additionally, a familiarization session was conducted, where a certified trainer assessed their back squat and bench press techniques, and their one-repetition maximum (1RM) for both exercises was determined.

For visits two through five, participants returned to the laboratory at the same time of day, performing four separate trials with at least 72 h between sessions to allow for full recovery and a washout period [8,20]. The four conditions tested included: (a) sodium bicarbonate combined with caffeine (NaHCO_3_ + CAF), (b) sodium bicarbonate alone (NaHCO_3_), (c) caffeine alone (CAF), and (d) a placebo (PLA). Each participant completed the trials in a randomized sequence, which was counterbalanced across the group using www.randomizer.org. An external researcher generated alphanumeric codes for participant identification and trial beverages to maintain blinding. These codes were only disclosed after the completion of statistical analyses.

### 2.3. Experimental Protocol

**Figure 1:**
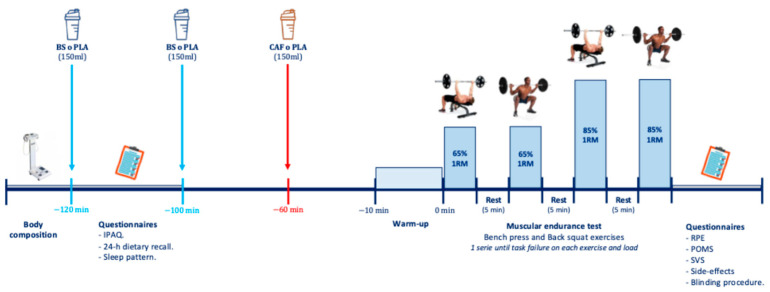
Experimental protocol.

The experimental procedure of the trial is illustrated in Figure 1 and explained below.

#### 2.3.1. Body Composition, Dietary and Physical Activity Habits

Participants attended a familiarization session in the laboratory one week before the initial experimental trial. Body composition was assessed using bioelectrical impedance analysis (Tanita BC-418, Tanita Corporation of America Inc., Chicago, IL, USA), as previously described in other studies [21], and body weight was utilized to determine individual supplement dosages. Dietary habits were analyzed using a 24 h dietary recall with MyFitnessPal software and DIAL (Alce Ingeniería, Madrid, Spain). Physical activity levels were measured using the International Physical Activity Questionnaire (IPAQ). Participants were instructed to abstain from consuming caffeine, stimulants, bicarbonate, alcohol, or engaging in intense physical exercise for 24 h leading up to the familiarization session and throughout the study period. Additionally, participants were required to maintain consistent dietary and sleep habits. Sleep patterns were self-reported through questionnaires, and diet and fluid intake were monitored using a 24 h dietary recall. Participants replicated their meals during the 24 h before each laboratory visit, particularly the two meals preceding the trials. To mitigate circadian variability, both familiarization and experimental trials were conducted at the same time of day.

#### 2.3.2. Supplementation Protocol

The supplementation process began 120 min prior to each trial. Participants ingested either sodium bicarbonate (0.3 g/kg body weight) or a placebo (3 mg/kg maltodextrin, HSN, Granada, Spain) in two doses of 0.15 g/kg (or 1.5 mg/kg for the placebo), administered 120 and 90 min before the session. This method was designed to reduce gastrointestinal discomfort [22]. Sixty minutes before the trial, participants consumed either caffeine (3 mg/kg, HSN, Granada, Spain) or a placebo (3 mg/kg maltodextrin). All supplements were dissolved in 150 mL of water and flavored with a non-caloric additive to mask taste and odor (MyProtein, Northwich, UK). To maintain blinding, the beverages were served in opaque shaker bottles.

#### 2.3.3. One Repetition Maximum (1RM)

The one-repetition maximum (1RM) for bench press and back squat exercises was assessed using a Smith machine (Multipower, Technogym, Barcelona, Spain), following protocols outlined in previous studies [23,24]. Participants were instructed to perform the eccentric phase in a controlled manner, pause for two seconds at the isometric phase, and then execute the concentric phase as quickly as possible. A linear transducer (Encoder, Chronojump Boscosystem, Collegno, Italy) measured the bar displacement velocity. Initial loads were set at 20 kg for males and 10 kg for females, increasing by 15–10 kg increments until the mean bar velocity (Vmean) reached 0.5 m/s for bench press and 0.8 m/s for back squat. To determine precise 1RM, smaller load increments (≤5 kg) were applied [23,24]. After a 20 min passive recovery period, participants completed a familiarization session, repeating the test sequence used during the experimental trials.

#### 2.3.4. Muscular Endurance Tests

Following a standardized warm-up consisting of 10 min of dynamic stretching and joint mobility exercises, participants performed muscular endurance tests [11,24]. These included one set to failure at 65% and 85% 1RM for both the bench press and back squat. The load and exercise sequence remained consistent across all trials, with a five-minute passive recovery period between exercises and load conditions. During each attempt, participants were instructed to control the eccentric phase, pause for two seconds in the isometric phase, and perform the concentric phase at maximum speed while maintaining a consistent movement range. A linear encoder (Encoder, Chronojump Boscosystem, Italy) recorded variables such as mean velocity (Vmean), peak velocity (Vpeak), time to peak velocity (time to Vpeak), mean power (Wmean), peak power (Wpeak), time to peak power (time to Wpeak), and rate of power development (RPD). The lowest repetition count was used for comparison across experimental conditions, with the averages of the recorded variables calculated for analysis.

#### 2.3.5. Questionnaires and Scales

Participants completed questionnaires at the end of the familiarization session and after each trial. These assessed perceived power, endurance, energy, muscular discomfort, gastrointestinal distress, rating of perceived exertion (RPE), heart rate, and other factors using a 1-to-10-point scale [25]. Mood was evaluated using an abbreviated version of the Profile of Mood States (POMS) and the Subjective Vitality Scale (SVS) [26]. To confirm the effectiveness of the blinding process, participants answered a specific question after each trial.

### 2.4. Statistical Analysis

The collected data were analyzed using SPSS version 27.0 (SPSS Inc., Chicago, IL, USA), and all visual representations were created with GraphPad Prism version 8 (GraphPad Software Inc., La Jolla, San Diego, CA, USA). Data normality was assessed using the Shapiro–Wilk test (*p* > 0.05). Muscular endurance performance was analyzed using a three-way repeated measures ANOVA based on supplement condition (NaHCO_3_ + CAF, NaHCO_3_, CAF, and PLA), load (65% and 85% 1RM), and exercise type (bench press and back squat). Sphericity was tested using Mauchly’s test, and violations were corrected using the Huynh-Feldt adjustment. The Holm-Bonferroni method was applied as a post hoc test where significant differences emerged. Cochran’s Q test was employed to analyze the effectiveness of blinding and supplement-related side effects.

Results are presented as mean ± standard deviation (SD), with statistical significance set at *p* < 0.05. Effect size (ES) was determined using partial eta squared (ηp^2^) for repeated measures ANOVA and Hedges’s g for pairwise comparisons. Effect size interpretations were defined as follows: trivial (0–0.19), small (0.20–0.49), medium (0.50–0.79), and large (≥0.80).

## 3. Results

No significant differences were found between the experimental conditions regarding body composition, dietary intake, or physical activity levels (Table 1).

### 3.1. Muscular Endurance

**Figure 2:**
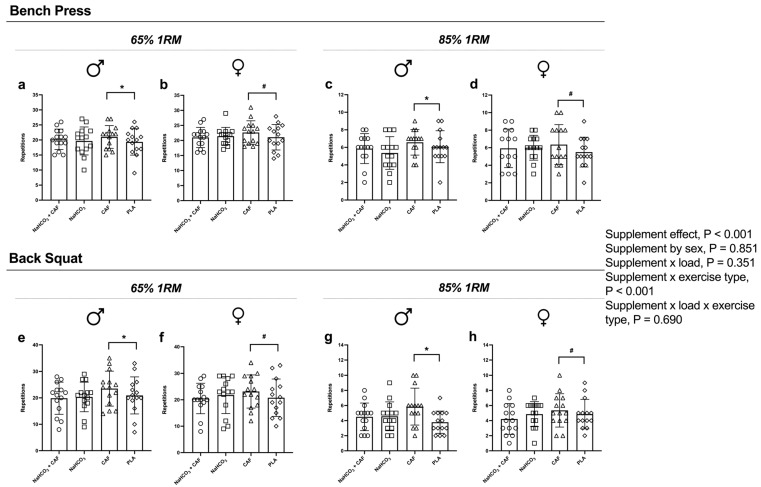
Number of repetitions performed after the four supplementation protocols at different intensities. Note: Number of repetitions performed in the bench press at 65%1RM in males (**a**) and females (**b**) and at 85%1RM in males (**c**) and females (**d**); and back squat exercise at 65%1RM in males (**e**) and females (**f**) and at 85%1RM in males (**g**) and females (**h**). * *p* < 0.05 CAF compared to PLA in male participants; # *p* < 0.05 CAF compared to PLA in female participants. Abbreviations: CAF, caffeine; NaHCO_3_, sodium bicarbonate; NaHCO_3_ + CAF, sodium bicarbonate plus caffeine; PLA, placebo.

**Figure 3:**
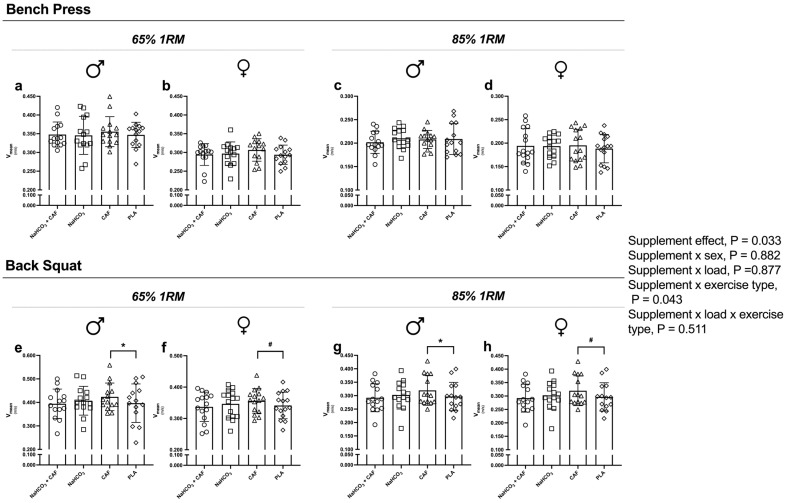
Mean velocity (V_mean_) performed after the four supplementation protocols at different intensities. Note: V_mean_ performed in the bench press at 65%1RM in males (**a**) and females (**b**) and at 85%1RM in males (**c**) and females (**d**); and back squat exercise at 65%1RM in males (**e**) and females (**f**) and at 85%1RM in males (**g**) and females (**h**). * *p* < 0.05 CAF compared to PLA in male participants; # *p* < 0.05 CAF compared to PLA in female participants. Abbreviations: CAF, caffeine; NaHCO_3_, sodium bicarbonate; NaHCO_3_ + CAF, sodium bicarbonate plus caffeine; PLA, placebo.

**Figure 4:**
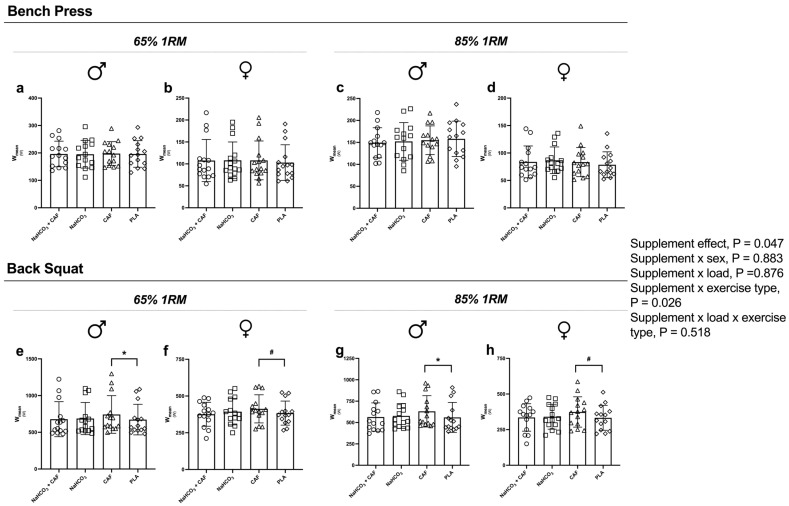
Mean power output (W_mean_) performed after the four supplementation protocols at different intensities. Note: W_mean_ performed in the bench press at 65%1RM in males (**a**) and females (**b**) and at 85%1RM in males (**c**) and females (**d**); and back squat exercise at 65%1RM in males (**e**) and females (**f**) and at 85%1RM in males (**g**) and females (**h**). * *p* < 0.05 CAF compared to PLA in male participants; # *p* < 0.05 CAF compared to PLA in female participants. Abbreviations: CAF, caffeine; NaHCO_3_, sodium bicarbonate; NaHCO_3_ + CAF, sodium bicarbonate plus caffeine; PLA, placebo.

Supplement effect was detected in the number of repetitions (Reps, *p* < 0.001; η_p_^2^ = 0.111; Figure 2), V_mean_ (*p* = 0.033; η_p_^2^ = 0.115; Figure 3), W_mean_ (*p* = 0.047; η_p_^2^ = 0.122; Figure 4) and W_peak_ (*p* = 0.044; η_p_^2^ = 0.106). Supplement plus exercise type (bench press vs. back squat) effect was detected in Reps (*p* < 0.001; η_p_^2^ = 0.109; Figure 2), V_mean_ (*p* = 0.033; η_p_^2^ = 0.115; Figure 3), W_mean_ (*p* = 0.026; η_p_^2^ = 0.120; Figure 4) and W_peak_ (*p* = 0.035; η_p_^2^ = 0.112). These effects were irrespective to sex or load.

Compared to placebo, caffeine intake improved Reps (*p* = 0.035, g = 0.22), V_mean_ (*p* = 0.043, g = 0.16) and Wmean (*p* = 0.034, g = 0.15) and Wpeak (*p* = 0.040, g = 0.17) in back squat exercise. At 65% 1RM, caffeine increased Reps by 7% (*p* = 0.048, g = 0.78) in bench press and back squat (*p* = 0.044, g = 0.63), while, at 85% 1RM, caffeine increased Reps by 9.5% (*p* = 0.050, g = 0.82) in bench press and by 25% (*p* = 0.045, g = 0.42) in back squat compared to placebo. In contrast, compared to placebo, a non-statistically significant difference was found in NaHCO_3_ + CAF and NaHCO_3_ in bench press at 65%1RM (5% and 4 %, respectively) and 85%1RM (2.5% and 3.8%, respectively) as well as in back squat exercise at 65%1RM (5.7% and 7%, respectively) and at 85%1RM (1% and 7%, respectively).

Moreover, compared to placebo, caffeine intake improved V_mean_ (3.7%, *p* = 0.050, g = 1.144) and W_mean_ (5.2%, *p* = 0.047, g = 0.986) in back squat at 65%1RM, while at 85%1RM caffeine increased V_mean_ by 5.4% (*p* = 0.043, g = 0.22) and W_mean_ by (5.5%, *p* = 0.050, g = 0.25), as well as V_peak_ by a 4.6% (*p* = 0.034, g = 0.23) and W_peak_ by 7.1% (*p* = 0.022, g = 0.19).

### 3.2. Side-Effects Derived from Supplements Trials

The side effects questionnaire demonstrated a significant interaction effect for gastrointestinal discomfort (*p* = 0.014, η_p_^2^ = 0.124) and muscular discomfort (*p* = 0.012, η_p_^2^ = 0.162) following the trials. Pairwise comparisons revealed that gastrointestinal discomfort was significantly greater in the NaHCO_3_ + CAF condition compared to CAF alone (41%; *p* = 0.011, g = 0.82) and the placebo (52%; *p* = 0.029, g = 1.01). Similarly, NaHCO_3_ alone increased gastrointestinal discomfort relative to CAF (39%; *p* = 0.042, g = 0.912) and the placebo (51%; *p* = 0.012, g = 1.126). In terms of perceived energy, participants reported higher levels in the NaHCO_3_ + CAF condition (21%; *p* = 0.021, g = 0.643) and in the CAF condition (17%; *p* = 0.039, g = 0.445) compared to the placebo.

No significant differences were observed in mood-related variables, including depression, anger, vigor, fatigue, confusion, tension, or the Subjective Vitality Scale (SVS). Finally, 66% of participants accurately identified when they consumed NaHCO_3_, while 55% correctly recognized when they ingested CAF.

## 4. Discussion

The purpose of this study was to evaluate acute co-ingestion of caffeine and NaHCO_3_ effect on muscular endurance according to load in bench press and back squat exercise in male and female resistance-trained participants. The main outcome of this investigation shows that caffeine intake increased the number of repetitions, V_mean_ and W_mean,_ particularly in back squat exercise compared to bench press exercise similarly in male and female participants; however, this performance-enhancing effect was not observed when caffeine was ingested together with NaHCO_3_ or when NaHCO_3_ was ingested alone. Thus, acute caffeine and NaHCO_3_ co-ingestion does not cause a synergic effect on muscular endurance; in fact, it mitigates the ergogenic effect caused by caffeine.

Muscular endurance is a vital attribute in resistance training and various sports disciplines, as it supports the ability to sustain force and power output against a given load over an extended duration. This study investigated the impact of acute co-ingestion of caffeine and sodium bicarbonate (NaHCO_3_) on muscular endurance. The tests lasted approximately 36 s for sets at 65% 1RM and around 14 s for sets at 85% 1RM. Previous meta-analyses and systematic reviews report that caffeine consumption can enhance muscular endurance by about 6–7% [27], primarily due to an increase in the total repetitions completed per set following acute caffeine ingestion. However, limited research has examined multiple performance parameters, such as power and velocity, during muscular endurance testing across different loads. Existing studies indicate that caffeine can increase repetitions, velocity, and power output at 85% 1RM during the bench press exercise [28]. This effect may be less pronounced for lower body exercises, such as back squats, which involve larger muscle groups [11]. The results of this study are consistent with prior findings. Compared to the placebo condition, caffeine supplementation improved the number of repetitions in both bench press and back squat exercises across 65% 1RM and 85% 1RM loads, with gains ranging from 7% to 25%. Additionally, caffeine enhanced mean velocity (Vmean) and mean power (Wmean) in the back squat at 65% 1RM (by 3.7% and 5.2%, respectively) and at 85% 1RM (by 5.4% and 5.5%, respectively). These benefits are likely linked to caffeine’s role in increasing calcium (Ca^2+^) release from the sarcoplasmic reticulum, as caffeine binds to ryanodine receptor 1 [29]. Enhanced Ca^2+^ release and reutilization in skeletal muscle may explain the observed increase in repetitions across exercises and loads. Moreover, caffeine is known to improve motor unit recruitment and the conduction velocity of muscle fibers, which varies depending on the size of the muscle group [30]. Research suggests that muscle activation during maximal voluntary contractions (MVC) is generally lower in larger muscle groups, such as the knee extensors, compared to smaller muscle groups, like the ankle plantar flexors [31]. Therefore, if caffeine’s ergogenic effects occur via central nervous system (CNS) stimulation, larger muscle groups may experience a more significant boost in force production due to increased motor unit recruitment and firing rates compared to smaller muscle groups. In contrast, if caffeine exerts a direct effect on muscle, its ergogenic benefits should be consistent across different muscle groups, regardless of size. However, prior research supports the hypothesis of a central effect, showing a more substantial improvement in variables like mean power (Wmean), peak power (Wpeak), and peak velocity (Vpeak) in larger muscle groups, such as the quadriceps (back squat), compared to smaller muscle groups, like the pectoralis (bench press) [11]. This study further supports these findings by comparing the ergogenic effects of a 3 mg/kg caffeine dose on the bench press and back squat exercises. The results demonstrated a greater improvement in mean velocity (Vmean), peak velocity (Vpeak), mean power (Wmean), and peak power (Wpeak) for the back squat, particularly at 85% 1RM. Acute caffeine ingestion appears to delay fatigue onset, enabling participants to complete more repetitions in both upper- and lower-body exercises. However, the increase in power and velocity production was more pronounced in the back squat, which involves larger muscle groups. This pattern suggests that caffeine’s ergogenic effects involve both central and peripheral mechanisms. Specifically, the central effects of caffeine appear to improve power and velocity based on muscle group size, while its peripheral effects enhance endurance by increasing the number of repetitions performed, regardless of muscle group size.

Sodium bicarbonate intake increased the number of repetitions by 3–8% in the loads (65% and 85%1RM) and exercise types (bench press and back squat) evaluated; however, these differences were not statistically significant. Previous studies reported equivocal evidence regarding NaHCO_3_ erogenicity on muscular endurance after ingesting 0.3 g/kg of NaHCO_3_ from 60 to 90 min before three sets of back squat and bench press exercises at 80%1RM in eight resistance-trained men [16] or wrist flexion exercise in six recreationally active men [32]. In contrast, other studies are aligned with our results, not showing an effect of 0.3 g/kg of NaHCO_3_ intake from 105 to 120 min before 10RM of the bench and pull press exercises in eleven resistance-trained men [33], five sets of leg press with loads adjusted to perform 12 repetitions in fifteen resistance-trained men [34] or 1 set of leg press at 70%1RM in six recreationally active men [35]. Since blood bicarbonate levels were not measured in this study, it can be suggested that a 5–6 mmol/L increase in blood bicarbonate was not reached and thus may explain the limited ergogenic effect found after NaHCO_3_ intake [36], although others have questioned this idea [14]. Another potential explanation for the lack of effect observed could be that the NaHCO_3_ erogenicity may be greater for exercises that activate small muscle groups [37] since higher blood flow occurs when these muscle groups are exercised, resulting in greater ion exchange. Nonetheless, no differences were found in this study when comparing the *quadriceps femoris* and *major pectoralis* as the main muscles involved in the back squat and bench press exercises, respectively. This suggests that muscle size group may not be a major factor in NaHCO_3_ erogenicity. Nonetheless, it cannot be avoided that NaHCO_3_ seems to be more effective in ≥30 s duration tasks consistent with the alkalosis induced in exercise lasting 1–10 min [38] and ergogenic effect that has been previously noted in other repeated efforts (i.e., repeated 30 s sprint tests) [39]. Therefore, future studies should explore resistance exercise protocols performing a higher number of sets (e.g., 3–4 sets) using a moderate load (e.g., 60–70% 1RM) that allows a sufficient effort duration (>30 s) with a rest interval between sets (e.g., ~1.5 min) that facilitates a pronounced reduction in pH levels to promote the buffering capacity of NaHCO_3_ [40].

The acute co-ingestion of NaHCO_3_ and caffeine did not lead to a significant improvement in the number of repetitions compared to placebo without altering mean velocity or power of force production. Although, to our knowledge, no previous studies analyzed the combined effect of NaHCO_3_ and caffeine on muscular endurance, some studies have utilized this supplement protocol in disciplines where muscular endurance is required, such as judo, where the synergic effect was found in ten male judokas who improved Special Judo Fitness Tests performance after ingesting 0.3 g/kg of NaHCO_3_ three days before the exercise session plus 0.1 g/kg the day of the testing in three occasions 120, 90 and 60 min and 6 mg/kg of caffeine 50 min before the trial [22].

However, this effect was not evident when administering caffeine doses of 3–6 mg/kg combined with 0.3 g/kg of sodium bicarbonate (NaHCO_3_), provided in one or multiple doses between 120 and 60 min before testing. Similar outcomes were reported in twelve elite rowers following a 6 min rowing test [41], ten trained cyclists during a 3 km time trial [42], thirteen untrained participants cycling to volitional exhaustion [43], and eight karate athletes performing a sport-specific aerobic test [44]. In this study, no additive effect was observed when consuming caffeine and NaHCO_3_; in fact, the ergogenic effect observed after caffeine ingestion in V_mean_ and W_mean_ was mitigated when this supplement was consumed together with NaHCO_3_. Peak plasma concentrations of caffeine normally occur between 45 and 120 min after oral ingestion; this variation in time may be dependent on gastric emptying time and the presence of other dietary constituents. Additionally, while caffeine stimulates gastric acid secretion [45], NaHCO_3_ neutralizes the increase in pH caused by gastric acid [46]. Collectively, a potential explanation for the lack of effect on NaHCO_3_ + CAF compared to the CAF condition observed may be attributable to the fact that NaHCO_3_ ingestion could delay caffeine absorption and bioavailability in blood when is co-ingested with NaHCO_3_. This may also explain, at least in part, the equivocal evidence found in the literature in studies providing acute caffeine and NaHCO_3_ co-ingestion with performance-enhancing purposes. Nonetheless, this idea required further studies to explore the pharmacokinetics and pharmacodynamics of these dietary supplements when consumed together.

A key limitation of this study was the inability to measure plasma concentrations of caffeine, bicarbonate, and pH. These measurements could have offered important insights into the absorption, effectiveness of the supplements, and any potential pharmacokinetic or pharmacodynamic interactions. Sodium bicarbonate could interact with caffeine by increasing gastric pH, which can alter caffeine’s solubility and absorption rate [47], by alkalinizing urine that may affect caffeine’s renal clearance and metabolism [48], and by affecting CYP1A2 enzyme activity through pH alterations, which could influence caffeine’s metabolism [49]. Additionally, another limitation was the lack of measurement of other blood markers (e.g., LDH or CK) that would increase the understanding of the fatigue caused by the exercise protocol performed in this study.

## 5. Conclusions

The combined intake of caffeine and sodium bicarbonate (NaHCO_3_) did not result in an additive ergogenic effect on muscular endurance performance. A key finding of this study was that caffeine alone enhanced the number of repetitions, average velocity, and power output in both male and female participants, with a more notable effect observed in the back squat compared to the bench press exercise. Although NaHCO_3_ did not improve muscular endurance performance, in fact, this substance mitigated caffeine’s ergogenic effect when both supplements were co-ingested. Therefore, based on the current results, we recommend the ingestion of caffeine but not combined with NaHCO_3_ before any sport where muscular endurance plays a critical role in performance (e.g., CrossFit or rowing).

## Figures and Tables

**Table 1:** Body composition, dietary, and physical activity habits in each experimental group.

	NaHCO_3_ + CAF	NaHCO_3_	CAF	Placebo	ANOVA *p*-Value (Partial η^2^)
	*Mean ± SD*	*Mean ± SD*	*Mean ± SD*	*Mean ± SD*	
*Body composition*					
Body mass (kg)	68.5 ± 13.3	68.3 ± 10.9	68.3 ± 13.2	68.2 ± 14.0	0.631 (0.113)
Fat mass (kg)	10.5 ± 4.9	10.9 ± 4.6	10.2 ± 4.3	10.4 ± 4.7	0.493 (0.150)
Fat-free mass (kg)	59.3 ± 11.2	59.6 ± 10.9	59.2 ± 11.1	59.1 ± 11.9	0.781 (0.040)
*Dietary habits*					
Energy intake (kcal)	1150 ± 310	1143 ± 333	1147 ± 319	1146 ± 320	0.612 (0.128)
Protein (g/kg)	1.2 ± 0.6	1.1 ± 0.4	1.1 ± 0.4	1.1 ± 0.5	0.752 (0.067)
Carbohydrate (g/kg)	1.9 ± 1.1	2.0 ± 1.1	2.0 ± 1.3	2.0 ± 1.2	0.847 (0.068)
Fat (g/kg)	0.60 ± 0.30	0.62 ± 0.30	0.65 ± 0.36	0.62 ± 0.32	0.365 (0.180)
*Physical Activity habits*					
METs-min/wk	10,543 ± 7145	10,590 ± 7243	10,556 ± 7224	10,515 ± 7114	0.645 (0.109)

Data are provided as mean ± standard deviation. The dietary habits reported represent the two meals ingested before the trials. Abbreviations: MET, metabolic equivalent of task.

## Data Availability

The original contributions presented in this study are included in this article; further inquiries can be directed to the corresponding authors.
